# Bridging theory and practice: a scoping review protocol on gamification’s impact in clinical reasoning education

**DOI:** 10.1136/bmjopen-2024-086262

**Published:** 2024-12-04

**Authors:** Ching-Yi Lee, Ching-Hsin Lee, Hung-Yi Lai, Po-Jui Chen, Mi-Mi Chen, Sze-Yuen Yau

**Affiliations:** 1 Department of Neurosurgery, Chang Gung Memorial Hospital Linkou Main Branch, Taoyuan, Taiwan; 2 Chang Gung University College of Medicine, Taoyuan, Taoyuan, Taiwan; 3 Department of Radiation Oncology, Proton and Radiation Therapy Center, Chang Gung Memorial Hospital at Linkou, Linkou, Taiwan; 4 Chang Gung Medical Education Research Centre, Taoyuan City, Taiwan

**Keywords:** Clinical Reasoning, EDUCATION & TRAINING (see Medical Education & Training), MEDICAL EDUCATION & TRAINING

## Abstract

**Abstract:**

**Introduction:**

In the rapidly evolving field of medical education, gamification has emerged as a promising strategy to enhance clinical reasoning skills among healthcare professionals. By incorporating game-like elements into the learning environment, gamification strives to enhance engagement, motivation and knowledge retention. Given the importance of clinical reasoning in medical decision-making and patient care, this scoping review protocol aims to systematically explore developments, implementations and outcomes of gamification in clinical reasoning education.

**Methods and analysis:**

The scoping review will follow the Arksey and O'Malley methodological framework, enhanced by guidelines from the Joanna Briggs Institute. We will search four major databases: OVID Medline, Scopus and Web of Science using key terms such as “gamification,” “clinical reasoning,” and “medical education”. Studies will be selected based on the participants, concepts and contexts (PCC) framework, focusing on literature published in English. Two independent reviewers will screen studies and extract data on gamification elements used in clinical reasoning education. Any disagreement between the reviewers will be resolved by consulting a third person. We will provide a narrative synthesis of the findings, highlighting the variety of gamified strategies and their effects on clinical reasoning skills. This review will also map out gaps in the current literature and provide direction for future research.

**Ethics and dissemination:**

The scoping review, which aggregates and synthesises publicly available studies, does not require ethics approval due to its nature as a compilation of existing research. The reporting of findings will adhere to the Preferred Reporting Items for Systematic Reviews and Meta-Analyses extension for Scoping Reviews (PRISMA-ScR) checklist, promoting both thoroughness and transparency in our analysis. Our dissemination plan encompasses publication in a peer-reviewed journal and presentations at academic conferences focused on medical education. This strategy is designed to engage educators, curriculum designers and policymakers within the sector, ensuring our insights reach those who can apply them most effectively.

**STRENGTHS AND LIMITATIONS OF THIS STUDY**

- This scoping review will use a rigorous methodology following the Arksey and O'Malley framework and Joanna Briggs Institute guidelines, ensuring a systematic and comprehensive approach to identifying and analysing studies related to gamification in clinical reasoning education.
- The inclusion of grey literature and non-peer-reviewed sources allows for a more inclusive overview of gamified strategies.
- The exclusion of non-English studies may limit the generalizability of the findings due to potential language bias.

## Introduction

 The landscape of medical education is witnessing a paradigm shift, fundamentally altering the pedagogical approaches to training the next generation of healthcare providers.^1 2^ This shift is not merely a reaction to the evolving landscape of patient care needs and healthcare delivery systems, but a proactive adaptation to the burgeoning complexity of global health challenges and the meteoric rise in medical technological advancements. In response to these changes, educators are exploring innovative teaching strategies that can better engage learners and enhance the development of critical skills such as clinical reasoning.^3^,^5^ Clinical reasoning, a complex cognitive process involving the synthesis and application of knowledge to diagnose and treat patients, stands at the heart of effective medical practice.^6 7^ However, traditional educational approaches, such as lectures and textbook-based learning, fall short in fully engaging learners or simulating the multifaceted nature of real-world medical decision-making.^8^

As medical educators grapple with these changes, the quest for innovative and effective teaching methodologies has brought gamification into the spotlight.^9^,^11^ Gamification, defined as the strategic incorporation of game-like elements and principles into non-gaming contexts, aims to transcend traditional educational boundaries.^11^,^14^ By embedding points, badges, leaderboards, challenges and storylines into the learning process, it taps into innate human desires for competition, achievement, recognition and collaboration.^10 11 14^ This educational strategy, with its roots in behavioural psychology and motivational theory, promises to redefine the acquisition and application of clinical skills and knowledge.^15^ The literature is replete with evidence supporting gamification’s positive impact on learning outcomes across a diverse array of disciplines, from science and mathematics to language acquisition, indicating its versatility and effectiveness.^16^,^18^

### Enhanced engagement

Gamification leverages interactive case studies and virtual patient scenarios, transforming passive learning into an active problem-solving adventure. This not only captivates learners’ attention but also fosters deeper engagement with the clinical content.^19^,^21^

#### Increased motivation

By integrating game mechanics like levels, badges and reward systems, learners are incentivised to progress through increasingly complex clinical reasoning tasks. This progression mimics the achievement system inherent in games, spurring motivation and dedication.^13 21^

#### Improved comprehension

The immediate feedback mechanism, a staple in gaming, allows learners to see the consequences of their decisions in real time, within a safe and controlled environment. This aspect is particularly beneficial in clinical reasoning, where understanding the cause-effect relationship is crucial.^22^,^24^

#### Immersive learning experience

By simulating real-life medical challenges, gamification provides a vividly immersive experience that prepares students for the high-stakes environment of healthcare. It enables learners to experience the pressure and urgency of medical decision-making, fostering resilience and adaptability.^13 22 25^

Given the instrumental role of clinical reasoning in ensuring patient safety and quality of care, the exploration of gamification’s efficacy in its education is both timely and critical. Traditional educational settings, with their limitations in fostering active learning and practical application, underscore the need for innovative approaches like gamification. The promise of gamification lies in its potential to bridge the gap between theoretical knowledge and real-world application, making the acquisition of complex clinical reasoning skills more accessible, engaging and effective.^11 19 26^

While previous reviews have examined gamification’s role in medical education broadly, there remains a gap in understanding its specific impact on clinical reasoning.^27^,^29^ A scoping review by Koelewijn *et al* provides valuable insights into the role of games in clinical reasoning education.^27^ However, the key difference between our study and that review lies in the focus: our study emphasises gamification, which incorporates game-like elements into non-game educational environments, whereas the previous review centres on serious games specifically designed for educational purposes. While our approach offers broader applicability in traditional educational settings, it may not provide the same level of immersion or engagement as fully developed games. Additionally, our review incorporates grey literature, ensuring a comprehensive analysis of both peer-reviewed and non-peer-reviewed sources. This scoping review endeavours to offer a comprehensive examination of the development, implementation and outcomes of gamification in clinical reasoning education. By synthesising existing evidence and insights from a broad spectrum of studies, it aims to illuminate the transformative power of gamification in medical education. The review seeks not only to assess the current state of gamified learning in clinical reasoning but also to identify gaps in the literature and directions for future research. Through this detailed exploration, the review aspires to contribute significantly to the ongoing discourse on enhancing medical education, with the ultimate goal of improving patient care and medical decision-making processes.

### Eligibility criteria

#### Participants

This scoping review will include studies involving a diverse range of participants engaged in clinical reasoning education across various healthcare fields. The review will target medical students, healthcare professional trainees, practitioners and paramedical professionals (eg, paramedics and respiratory therapists) who participate in or benefit from gamified learning interventions designed to enhance clinical reasoning skills. Our goal is to highlight the varied applications and effects of gamification strategies throughout healthcare education, acknowledging clinical reasoning’s interdisciplinary character. Consequently, we will include all healthcare professionals who use gamification in clinical reasoning training, ensuring our review benefits from inclusive perspectives across a wide array of clinical environments and educational scenarios.

#### Concepts

This scoping review explores the impact of gamification on clinical reasoning education within various healthcare fields. It examines how integrating game-like elements and mechanics, such as points, levels, challenges and immediate feedback, into educational strategies enhances engagement, motivation, comprehension and importantly, the clinical reasoning skills of healthcare professionals.^11^,^14^ In this broader context, serious games, which are fully developed, game-based environments specifically designed for educational purposes, are also considered part of gamification, as they use game mechanics to facilitate learning.^9 28^ These serious games often immerse learners in simulated clinical scenarios that challenge their clinical reasoning skills, providing structured goals and feedback to enhance learning outcomes. However, simulations, which recreate real-life clinical environments where learners practice clinical skills in a controlled setting, are not included within the scope of gamification.^30 31^ Therefore, simulations focus only on replicating real-world tasks rather than using game-like elements for educational engagement will be excluded from the analysis.

#### Context

The review will concentrate on research carried out in medical education environments that use gamification as a pedagogical tool for clinical reasoning. These environments may comprise classrooms, online learning platforms, simulation laboratories and clinical rotations, among others. There will be no geographical restrictions; pertinent studies from across the globe will be considered to encompass a broad spectrum of applications and outcomes. Nevertheless, studies not centred on the medical domain or the enhancement of clinical reasoning education through gamification will be excluded.

### Types of sources

The review will incorporate mixed-methods study designs to facilitate a comprehensive exploration of the subject matter. This will include experimental studies (such as randomised controlled trials and quasi-experimental studies), observational studies (such as cohort, cross-sectional and case-control studies) and qualitative research (such as interviews, focus groups and thematic analyses) that offer insights into the adoption and impact of gamification in clinical reasoning education. Furthermore, systematic reviews that aggregate findings on gamification within this educational sphere will be examined.

Both peer-reviewed scientific articles and pertinent grey literature, including editorials, commentaries and brief reports, will be considered to capture a wide scope of discussion on gamification in clinical reasoning education comprehensively. Conversely, non-peer-reviewed book chapters and conference proceedings will be omitted due to possible constraints in methodological soundness and evidence quality. Primarily, studies published in English will be included to guarantee the accessibility and intelligibility of the findings, with selective incorporation of research in other languages contingent on the availability of translations and their pertinence to the review’s goals.

## Methods

This scoping review will adhere to the structured approach as detailed by the Joanna Briggs Institute (JBI) methodology and the framework set by Arksey and O'Malley for conducting scoping reviews.^32 33^ The Preferred Reporting Items for Systematic Reviews and Meta-Analyses extension for Scoping Reviews (PRISMA-ScR) guidelines will be followed to ensure a systematic and transparent approach not only to the flow diagram but also throughout the entire review process,^34^ including accurately documenting the identification, screening, eligibility and inclusion of studies. The full ScR checklist will be applied to guarantee that all aspects of the review, from study selection to reporting of results, comply with established best practices for scoping reviews. The methodology unfolds across a systematic six-phase sequence: (1) identifying the research questions; (2) identifying studies; (3) selecting studies; (4) charting the data; (5) summarising and reporting the findings and (6) an optional consultation phase.

### (1) Identifying research questions: review questions

1. How is gamification developed and applied in the education of clinical reasoning across various healthcare professions?
2. What outcomes have been reported from using gamification strategies on the clinical reasoning skills of healthcare professionals?
3. Which elements of gamification are effective in advancing clinical reasoning education for healthcare professionals?
4. What are the theoretical underpinnings that inform the use of game elements in clinical reasoning education across healthcare professions?

### (2) Identifying studies: search strategy

The search strategy is crafted to extensively locate both published and unpublished (grey) literature on gamification in clinical reasoning education. This review will include grey literature, defined as non-peer-reviewed materials such as reports, white papers, government publications and theses. The inclusion of grey literature allows for a more comprehensive exploration of gamification strategies, especially those not captured in traditional peer-reviewed sources.

Initially, a preliminary search on MEDLINE (PubMed) and CINAHL (EBSCO) will focus on articles related to gamification in clinical reasoning education. A sophisticated search strategy, detailed in online supplemental S1, will be developed, using keywords and phrases identified in the titles and abstracts of relevant studies, along with their index terms. This search strategy will be tailored for each of three databases (OVID Medline, Scopus and Web of Science) to include all pertinent keywords and index terms identified in the initial search. The review will focus on studies published from 2012 onwards, reflecting the period when game-related publications in health education began to develop significantly.^27^

The search will then broaden to encompass additional databases pertinent to medical and educational research, adjusting the search terms to gather the broadest range of relevant studies. Additionally, the reference lists of all selected studies will be scrutinised to uncover more works meeting the inclusion criteria, aiming for a comprehensive dataset on gamification’s impact on clinical reasoning education.

### (3) Study/source of evidence selection

Identified citations will be organised and managed with EndNote 20 (Clarivate Analytics, PA, USA), which aids in eliminating duplicates. Additionally, Rayyan (Qatar Computing Research Institute, Doha, Qatar) will be used to further deduplicate and facilitate the selection of articles. After a pilot screening to fine-tune the selection criteria, titles and abstracts will be reviewed by two team members independently, using pre-set inclusion criteria. Any discrepancies will be resolved through discussion with a third reviewer. JBI SUMARI will assist in accessing full-text articles and managing citations. Potentially relevant articles will be closely examined for eligibility by two independent reviewers, with a detailed rationale for exclusions documented. Disagreements at any stage will be collaboratively resolved. The selection process, search results and inclusion details will be illustrated in a PRISMA-ScR flow diagram.^34^ This scoping review is planned to begin in October 2024 and is expected to be completed by April 2024. Data extraction, analysis and synthesis will occur within this period, ensuring the review is conducted systematically and within the specified timeframe.

### (4) Data extraction

Data from included studies will be extracted independently by two reviewers using a custom-designed extraction tool, devised after critical team discussions (online supplemental S2). The game elements will be listed according to a predefined list to ensure consistency and uniformity across studies.^35^ This predefined list includes elements including Achievements, Avatars, Badges, Bars, Competition, Experience Points, Feedback, Leaderboards, Levels, Narrative, Points, Quests/Goals/Challenges, Social, Stages and Time. Extracted data will encompass details about participants (levels of healthcare professionals), concepts (gamification elements and their design in clinical reasoning education), contexts (study settings, educational environments), methodologies and key findings pertinent to the review’s aims. An inductive thematic approach will guide data coding, starting with a subset of texts to identify emerging themes, followed by the application of a coding scheme across all included literature. Any conflicts in data extraction will be discussed among the team, with a third reviewer available for consultation. Authors might be contacted for further details or clarifications.

### (5) Data analysis and presentation

Data will be analysed and presented through a combined quantitative and thematic method within an analytical framework. The coding of each article will highlight up to two primary themes, facilitated by JBI SUMARI, with results summarised using this tool. Nominal data will be presented in percentages to illustrate the prevalence and patterns of gamification strategies in clinical reasoning education.

### (6) Consultation exercise

In line with best practices, the final phase will involve consultations with experts in gamification, medical education and clinical reasoning to include a range of perspectives and cultural considerations, thereby enriching the review’s outcomes. Expert input will be sought from specialists in medical education and public health, notably from leading institutions recognised for their educational innovation and research contributions.

### Ethics and dissemination

The scoping review, which aggregates and synthesises publicly available studies, does not require ethics approval due to its nature as a compilation of existing research. The reporting of findings will adhere to the PRISMA-ScR checklist, promoting both thoroughness and transparency in our analysis. Our dissemination plan encompasses publication in a peer-reviewed journal and presentations at academic conferences focused on medical education. This strategy is designed to engage educators, curriculum designers and policymakers within the sector, ensuring our insights reach those who can apply them most effectively.

## supplementary material

10.1136/bmjopen-2024-086262 online supplemental file 1

10.1136/bmjopen-2024-086262 online supplemental file 2
