# Supplementary material for: Bridging theory and practice: a scoping review protocol on gamification’s impact in clinical reasoning education
Source: BMJ Open. 2024 Dec 4;14(12):e086262. doi: 10.1136/bmjopen-2024-086262 (PMC11624824; doi:10.1136/bmjopen-2024-086262)
Supplement: online supplemental file 1 [file bmjopen-14-12-s001.docx]

# Supplement S1: Search Strategy for Retrieving Relevant Studies from 2012 Onwards Across Three Databases

## S1.1.

| **Database** | **Medline+Journals @ Ovid®** |
| --- | --- |
| **Search Results** | 1. "medical educat*".mp. 2. "medical train*".mp. 3. "medical teach*".mp. 4. "medical student*".mp. 5. (doctor* and (educat* or student* or train*)).mp. 6. (physician* and (educat* or student* or train*)).mp. 7. (surgeon* and (educat* or student* or train*)).mp. 8. (psychiatrist* and (educat* or student* or train*)).mp. 9. (dent* and (educat* or student* or train*)).mp. 10. (psycholog* and (educat* or student* or train*)).mp. 11. ("occupational therapist*" and (educat* or student* or train*)).mp. 12. ("physical therapist*" and (educat* or student* or train*)).mp. 13. ((dietitian* or nutritionist*) and (educat* or student* or train*)).mp. 14. (nurse* and (educat* or student* or train*)).mp. 15. (pharmac* and (educat* or student* or train*)).mp. 16. ("medical laboratory scientist*" and (educat* or student* or train*)).mp. 17. ("social worker*" and (educat* or student* or train*)).mp. 18. ("speech pathologist*" and (educat* or student* or train*)).mp. 19. ("respiratory therapist*" and (educat* or student* or train*)).mp. 20. (radiographer* and (educat* or student* or train*)).mp. 21. (optometrist* and (educat* or student* or train*)).mp. 22. OR / 1-21 23. reasoning.mp. 24. "problem solving".mp. 25. "decision making".mp. 26. OR/ 23-25 27. game*.mp 28. gamif*.mp. 29. OR/ 27–28 30. 22 AND 26 AND 29 31. limit 30 to (yr="2012 - 2024") 32. limit 31 to (english language) |

## S1.2.

| **Database** | **Scopus** |
| --- | --- |
| **Search Results** | 1. TITLE-ABS-KEY("medical educat*") 2. TITLE-ABS-KEY("medical train*") 3. TITLE-ABS-KEY("medical teach*") 4. TITLE-ABS-KEY("medical student*") 5. TITLE-ABS-KEY ( doctor* AND ( educat* OR student* OR train* )) 6. TITLE-ABS-KEY(physician* AND ( educat* OR student* OR train* )) 7. TITLE-ABS-KEY(surgeon* AND ( educat* OR student* OR train* )) 8. TITLE-ABS-KEY(psychiatrist* AND ( educat* OR student* OR train* )) 9. TITLE-ABS-KEY(dent* AND ( educat* OR student* OR train* )) 10. TITLE-ABS-KEY(psycholog* AND ( educat* OR student* OR train* )) 11. TITLE-ABS-KEY("occupational therapist*" AND ( educat* OR student* OR train* )) 12. TITLE-ABS-KEY("physical therapist*" AND ( educat* OR student* OR train* )) 13. TITLE-ABS-KEY((dietitian* OR nutritionist*) AND ( educat* OR student* OR train* )) 14. TITLE-ABS-KEY(nurse* AND ( educat* OR student* OR train* )) 15. TITLE-ABS-KEY(pharmac* AND ( educat* OR student* OR train* )) 16. TITLE-ABS-KEY("medical laboratory scientist*" AND ( educat* OR student* OR train* )) 17. TITLE-ABS-KEY ( "social worker*" AND ( educat* OR student* OR train* )) 18. TITLE-ABS-KEY ( "speech pathologist*" AND ( educat* OR student* OR train* )) 19. TITLE-ABS-KEY ( "respiratory therapist*" AND ( educat* OR student* OR train* )) 20. TITLE-ABS-KEY ( radiographer* AND ( educat* OR student* OR train* )) 21. TITLE-ABS-KEY ( optometrist* AND ( educat* OR student* OR train* )) 22. OR / 1-21 23. TITLE-ABS-KEY (reasoning) 24. TITLE-ABS-KEY("problem solving") 25. TITLE-ABS-KEY("decision making ") 26. OR/ 23-25 27. TITLE-ABS-KEY(game*) 28. TITLE-ABS-KEY(gamif*) 29. OR/ 27–28 30. 22 AND 26 AND 29 31. LIMIT 30 TO ( PUBYEAR >2011 AND PUBYEAR <2025 ) AND ( LANGUAGE , "English" ) |

## S1.3.

| **Database** | **Web of Science^TM^ (All Databases)** |
| --- | --- |
| **Search Results** | 1. TOPIC: (“medical educat*”) 2. TOPIC: (“medical train*”) 3. TOPIC: (“medical teach*”) 4. TOPIC: (“medical student*”) 5. TOPIC: (doctor* AND (educat* or student* or train*)) 6. TOPIC: (physician* AND (educat* or student* or train*)) 7. TOPIC: (surgeon* AND (educat* or student* or train*)) 8. TOPIC: (psychiatrist* AND (educat* or student* or train*)) 9. TOPIC: (dent* AND (educat* or student* or train*)) 10. TOPIC: (psycholog* AND (educat* or student* or train*)) 11. TOPIC: (“occupational therapist*” AND (educat* or student* or train*)) 12. TOPIC: (“physical therapist*” AND (educat* or student* or train*)) 13. TOPIC: ((dietitian* or nutritionist*) AND (educat* or student* or train*)) 14. TOPIC: (nurse* AND (educat* or student* or train*)) 15. TOPIC: (pharmac* AND (educat* or student* or train*)) 16. TOPIC: (“medical laboratory scientist*” AND (educat* or student* or train*)) 17. TOPIC: (“social worker*” AND (educat* or student* or train*)) 18. TOPIC: (“speech pathologist*” AND (educat* or student* or train*)) 19. TOPIC: (“respiratory therapist*” AND (educat* or student* or train*)) 20. TOPIC: (radiographer* AND (educat* or student* or train*)) 21. TOPIC: (optometrist* AND (educat* or student* or train*)) 22. OR / 1-21 23. TOPIC: (reasoning) 24. TOPIC: ("problem solving") 25. TOPIC: ("decision making ") 26. OR/ 23-25 27. TOPIC: (game*) 28. TOPIC: (gamif*) 29. OR/ 27–28 30. 22 AND 26 AND 29 31. Refined 30 by: PUBLICATION YEARS: ( 2012 OR 2013 OR 2014 OR 2015 OR 2016 OR 2017 OR 2018 OR 2019 OR 2020 OR 2021 OR 2022 OR 2023 OR 2024) AND LANGUAGES: ( ENGLISH) |
