# Supplementary material for: Bridging theory and practice: a scoping review protocol on gamification’s impact in clinical reasoning education
Source: BMJ Open. 2024 Dec 4;14(12):e086262. doi: 10.1136/bmjopen-2024-086262 (PMC11624824; doi:10.1136/bmjopen-2024-086262)
Supplement: online supplemental file 2 [file bmjopen-14-12-s002.docx]

# Supplement S2: Data Extraction Form

| Citation details | | | | | |
| --- | --- | --- | --- | --- | --- |
| Title | Authors | Publication date | Country of publication | Journal | Publication Type |
|  |  |  |  |  |  |
|  |  |  |  |  |  |
| Study Demographics | | | | | |
| Participants  Demographics | Specialities and levels of healthcare professionals | Sample Size | Study Setting | Research design | Study measures |
|  |  |  |  |  |  |
|  |  |  |  |  |  |
| Game-likes elements (Achievements, Avatar, Badges, Bars, Competition, Experience Points, Feedback, Leaderboard, Levels, Narrative, Points, Quests/Goals/Challenges, Social, Stages, Time. Etc.) | | | | | |
| Mechanisms | | | | | |
| Outcomes | | | | | |
