# Supplementary material for: Bridging theory and practice: a scoping review protocol on gamification’s impact in clinical reasoning education
Source: BMJ Open. 2024 Dec 4;14(12):e086262. doi: 10.1136/bmjopen-2024-086262 (PMC11624824; doi:10.1136/bmjopen-2024-086262)
Supplement: Supplementary file 3 [file bmjopen-2024-086262.reviewer_comments.pdf]

PEER REVIEW HISTORY

BMJ Open publishes all reviews undertaken for accepted manuscripts. Reviewers are asked to complete a checklist review form and are provided with free text boxes to elaborate on their assessment. These free text comments are reproduced below.

ARTICLE DETAILS

Title (Provisional)

Bridging Theory and Practice: A Scoping Review Protocol on Gamification's Impact in Clinical Reasoning Education

Authors

Lee, Ching-Yi; Lee, Ching-Hsin; Lai, Hung-Yi; Chen, Po-Jui; Chen, Mi-Mi; Yau, Sze-Yuen

---

VERSION 1 - REVIEW

**Reviewer**	**1**
**Name**	**Ang, Eng-Tat**
**Affiliation**	**National University of Singapore, Anaotmy**
**Date**	**01-Apr-2024**
**COI**	**Nil**

I am not entirely clear what is meant by grey literature?

If my mandarin is not good enough to understand the primary research, then I will have to reply on this review for the analysis/ conclusion?

Is this what you are saying? What about non English or Chinese literature? As mentioned, this is a major challenge and cannot be excluded.

I am not familiar with the Artriti library too.

Apologies, I cannot be more positive about this proposed research protocol.

**Reviewer**	**2**
**Name**	**Button, Dale**
**Affiliation**	**Durham College, Health Sciences**
**Date**	**23-Apr-2024**
**COI**	**None**

The process outlined here is complete. A suggestion: a more comprehensive list of health care professionals could be utilized in the search criteria. Paramedical providers are likely to be missed with the criteria listed.

**Reviewer**	**3**
**Name**	**Aster, Alexandra**
**Affiliation**	**University of Bonn**
**Date**	**24-Apr-2024**
**COI**	**No competing interests**

Throughout the protocol, specify the learning environments where gamification is studied. Attention should be given to distinguishing between gamification, serious games, and simulations. As outlined in the introduction, gamification is employed in non-gaming contexts, necessitating careful delineation of these contexts during data extraction to prevent contamination of data. Consistency in terminologies such as "game-like elements" and "game-design elements" is essential.

Data extraction sheet:

Clearly articulate empirical findings regarding the efficacy of game elements.

Determine whether game elements are listed according to a predefined list or cataloged without structure and classified subsequently. Consider incorporating the theoretical underpinnings of game elements.

"Encompass a diverse study design" in the Types of Sources section may be better replaced with "mixed-methods" for clarity. Clarify the meaning of "additional inclusion and exclusion criteria" and assess whether these criteria can be integrated with the previously mentioned criteria.

Methods:

Reference the PRISMA-ScR Checklist, emphasizing adherence not only to the flow diagram but also to the entire review process, as evidenced by the inclusion of the ScR Checklist.

Consider formulating a research question to explore the theoretical underpinnings of game elements.

Provide detailed information on how the search is broadened. Utilize tools such as Rayyan for the initial phase of abstract screening.

Determine if a third mediator is employed for resolving disagreements among reviewers.

VERSION 1 - AUTHOR RESPONSE

<u>Reviewer 1's Comments</u>

Point 1: I am not entirely clear what is meant by grey literature?
Response 1: The manuscript has been updated to clarify what is meant by grey literature, which includes non-peer-reviewed materials such as reports, theses, white papers, and government publications. This inclusion ensures a more comprehensive exploration of gamification strategies.

Point 2: If my mandarin is not good enough to understand the primary research, then I will have to reply on this review for the analysis/ conclusion?
Is this what you are saying? What about non English or Chinese literature? As mentioned, this is a major challenge and cannot be excluded. I am not familiar with the Artriti library too.
Response 2: Thank you for your insightful feedback. We originally designed this protocol with consideration of our language capacity, aiming to include studies published in both English and Chinese to capture relevant research. However, we acknowledge the reviewers' concerns regarding the accessibility of the primary research for researchers and readers who may not be proficient in Mandarin. In response to this feedback, we have revised the protocol to focus solely on the inclusion of English-language literature. As a result, we have eliminated the Chinese-language databases, including the Airiti Library, from our search strategy. This adjustment ensures that the findings of our review remain accessible and interpretable to a broader international audience, addressing potential language-related limitations. We believe this change will enhance the relevance and utility of our review for a wider group of researchers, while maintaining the integrity of the study.

<u>Reviewer 2's Comments</u>
Point 1: A suggestion: a more comprehensive list of health care professionals could be utilized in the search criteria. Paramedical providers are likely to be missed with the criteria listed.
Response 1: The search criteria have been expanded to include a broader range of healthcare professionals, including paramedics, respiratory therapists, and other allied health professionals, ensuring a more comprehensive scope and inclusion of paramedical providers.

<u>Reviewer 3's Comments</u>
Point 1: Throughout the protocol, specify the learning environments where gamification is studied. Attention should be given to distinguishing between gamification, serious games, and simulations. As outlined in the introduction, gamification is employed in non-gaming contexts, necessitating careful delineation of these contexts during data extraction to prevent contamination of data.
Response 1: The manuscript has been revised to clearly distinguish between gamification, serious games, and simulations. In the Concepts section, the distinction is made that gamification incorporates game-like elements into non-game educational contexts, while serious games are fully developed game environments. Simulations, which focus on replicating real-world clinical environments, are explicitly excluded from the analysis to avoid data contamination.

Point 2: Consistency in terminologies such as "game-like elements" and "game-design elements" is essential.
Response 2: Terminology consistency has been ensured throughout the manuscript, particularly in the use of terms like game-like elements and game-design elements. This is applied consistently across sections to avoid confusion.

Point 3: Clearly articulate empirical findings regarding the efficacy of game elements.
Determine whether game elements are listed according to a predefined list or cataloged without structure and classified subsequently. Consider incorporating the theoretical underpinnings of game elements.
Response 3: The data extraction process is detailed in the Methods section, clearly articulating how game elements will be cataloged. It specifies that game elements will be categorized according to predefined lists, and their empirical efficacy will be articulated based on the findings.

Point 4: "Encompass a diverse study design" in the Types of Sources section may be better replaced with "mixed-methods" for clarity. Clarify the meaning of "additional inclusion and exclusion criteria" and assess whether these criteria can be integrated with the previously mentioned criteria.
Response 4: The phrasing "encompass a diverse study design" has been replaced with "mixed-methods" for clarity. Additional inclusion/exclusion criteria have been integrated with the main inclusion/exclusion criteria to provide coherence.

Point 5: Reference the PRISMA-ScR Checklist, emphasizing adherence not only to the flow diagram but also to the entire review process, as evidenced by the inclusion of the ScR Checklist.
Response 5: The PRISMA-ScR Checklist has been referenced throughout the manuscript, ensuring adherence to best practices not only for the flow diagram but also across the entire review process.

Response 6: Consider formulating a research question to explore the theoretical underpinnings of game elements.
Response 6: We appreciate the reviewer's suggestion and have incorporated a specific research question to explore the theoretical foundations that guide the use of game elements in clinical reasoning education. The new research question is as follows:
"*What are the theoretical underpinnings that inform the use of game elements in clinical reasoning education across healthcare professions?*"

Point 7: Provide detailed information on how the search is broadened. Utilize tools such as Rayyan for the initial phase of abstract screening. Determine if a third mediator is employed for resolving disagreements among reviewers.
Response 7: The search strategy is explained in detail, and the use of Rayyan for deduplication and abstract screening has been highlighted. The role of a third reviewer to resolve any disagreements is also included to ensure a rigorous and transparent selection process.

VERSION 2 - REVIEW

**Reviewer**	**3**
**Name**	**Aster, Alexandra**
**Affiliation**	**University of Bonn**
**Date**	**18-Oct-2024**
**COI**	

The reviewer comments were realized very well, and I would therefore recommend accepting the study protocol. I wish the authors great success in conducting the study.
